# Mutagenesis of GPR139 reveals ways to create gain or loss of function receptors

**DOI:** 10.1002/prp2.466

**Published:** 2019-02-07

**Authors:** Lien Wang, Grace Lee, Amy Shih, Chester Kuei, Diane Nepomuceno, Michelle Wennerholm, Frances Fan, Jiejun Wu, Pascal Bonaventure, Timothy W. Lovenberg, Changlu Liu

**Affiliations:** ^1^ Janssen Research & Development, LLC San Diego California; ^2^ Present address: UCSF Helen Diller Family Comprehensive Cancer Center San Francisco California

**Keywords:** calcium mobilization assay, gain of function, GPR139, homology model, random mutagenesis, reduction of function

## Abstract

GPR139 is a Gq‐coupled receptor activated by the essential amino acids L‐tryptophan (L‐Trp) and L‐phenylalanine (L‐Phe). We carried out mutagenesis studies of the human GPR139 receptor to identify the critical structural motifs required for GPR139 activation. We applied site‐directed and high throughput random mutagenesis approaches using a double addition normalization strategy to identify novel GPR139 sequences coding receptors that have altered sensitivity to endogenous ligands. This approach resulted in GPR139 clones with gain‐of‐function, reduction‐of‐function or loss‐of‐function mutations. The agonist pharmacology of these mutant receptors was characterized and compared to wild‐type receptor using calcium mobilization, radioligand binding, and protein expression assays. The structure‐activity data were incorporated into a homology model which highlights that many of the gain‐of‐function mutations are either in or immediately adjacent to the purported orthosteric ligand binding site, whereas the loss‐of‐function mutations were largely in the intracellular G‐protein binding area or were disrupters of the helix integrity. There were also some reduction‐of‐function mutations in the orthosteric ligand binding site. These findings may not only facilitate the rational design of novel agonists and antagonists of GPR139, but also may guide the design of transgenic animal models to study the physiological function of GPR139.

**Abbreviations**
ACTH: adrenocorticotropic hormone
DMSO: dimethyl sulfoxide
DPBS: Dulbecco's phosphate buffered saline
ECL: extra cellular loop
FBS: fatal bovine serum
FLIPR: fluorometric imaging plate reader
GPCR: G‐protein coupled receptors
HBSS: Hank's balanced salt solution
HPLC: high‐performance liquid chromatography
HRP: horseradish peroxidase
MD: molecular dynamics
MPP+: 1‐methyl‐4‐phenylpyridinium
PBS: phosphate buffered saline
TMB: 3,3’,5,5’‐tetramethylbenzidine
TM: transmembrane domain
α‐MSH, and β‐MSH: α‐, and β‐melanocyte stimulating hormone

## 1 INTRODUCTION

GPR139, aka GPRg1 or GPCR12, is a highly conserved Gq‐coupled receptor that belongs to the rhodopsin‐like family of G‐protein coupled receptors (GPCR).^1^GPR139 is almost exclusively expressed in central nervous system^2^, ^3^ where it is abundantly expressed in the medial habenula, caudate putamen and lateral septum, and is also detected in pituitary, however with much higher levels in rat compared to human.^1^ We and others have established that GPR139 is activated by the essential amino acids L‐tryptophan (L‐Trp) and L‐phenylalanine (L‐Phe) with EC_50_ values in the 30‐ to 300‐μmol L^−1^ range, which is consistent with the physiologic concentrations of both amino acids.^1^, ^4^ Definitive proof that these are the true endogenous ligands is, however, lacking due to the challenges of manipulating the levels of these essential amino acids in vivo without impacting other critical functions such as protein synthesis and neurotransmitter precursors.

Several groups have reported surrogate ligands for GPR139, including TC‐O 9311 (3,5‐dimethoxybenzoic acid 2‐[(1‐naphthalenylamino)carbonyl]hydrazide) and JNJ‐63533054 ((S)‐3‐bromo‐5‐chloro‐N‐(2‐oxo‐2‐((1‐phenylethyl)amino)ethyl)benzamide) as potent and selective agonists.^5^, ^6^, ^7^, ^8^ We also demonstrated that L‐Trp and L‐Phe activated GPR139‐dependent mitogen activated protein kinase‐ERK phosphorylation.^1^ Recently, Nøhr et al reported that GPR139 can be activated by adrenocorticotropic hormone (ACTH), α‐, and β‐melanocyte stimulating hormone (α‐MSH, and β‐MSH).^9^ However, our recent data do not support that GPR139 is activated by ACTH, α‐MSH, and β‐MSH at physiologically relevant concentrations. But we were able to demonstrate an in vitro interaction between GPR139 and the melanocortin receptors (MCRs, also known as melanocyte stimulating hormone receptors), which may be an artifactual result of recombinant expression.^10^

The physiological role of GPR139 is still poorly understood as it not clear yet whether L‐Trp and/or L‐Phe are the physiologically endogenous ligands nor is it clear whether the receptor exists to detect increasing or decreasing amino acid levels. There are limited in vivo studies utilizing the few tool compounds available. The GPR139 agonist JNJ‐63533054 induced a dose‐dependent reduction in locomotor activity in rats.^1^ Bayer Andersen et al^11^ have recently reported that the GPR139 agonist TC‐O 9311 protected primary mesencephalic dopamine neurons against 1‐methyl‐4‐phenylpyridinium (MPP+)‐mediated degeneration, indicating a potential role of GPR139 in neuroprotection and Parkinson's disease.^11^ Another GPR139 agonist, 4‐oxo‐3,4‐dihydro‐1,2,3‐benzotriazine, has been reported to improve social withdrawal.^12^

From a structural perspective, Kaushik and Sahi reported a three dimensional structure prediction and molecular dynamics simulation of GPR139 model. Further structure‐based virtual screening was applied and several active site residues of GPR139, including Tyr32, Glu105, Glu108, and Tyr192, have been identified as potential ligand binding sites for inhibition of protein dimerization and receptor activity.^13^ Shehata et al^14^ also presented a combined structure‐activity relationship, and a refined pharmacophore model of GPR139.^14^ By applying in vitro and in silico mutagenesis, they demonstrated a common binding site for GPR139 surrogate agonists, including TC‐O 9311, JNJ‐63533054, L‐Trp, and L‐Phe, indicating that residues Phe109, His187, and Asn271 are important for the binding of these ligands.^15^

The goal of the present study was to further understand the critical structural motifs for GPR139 receptor activity as well as create new tools to further study the physiological role of GPR139. We used both site‐directed and random mutagenesis approaches and identified series of human GPR139 mutation clones with gain‐of‐function, reduction‐of‐function as well as loss‐of‐function properties. The pharmacological profiles of these mutant receptors were characterized by using calcium mobilization assay, radioligand binding assays and receptor protein expression assay. Further, these structure‐activity data were incorporated into a homology model. Collectively, the results suggest that the endogenous L‐Trp, and L‐Phe share a common binding site with the GPR139 agonists, TC‐O 9311 and JNJ‐63533054. This common binding site consists of a buried hydrophobic pocket defined by residues Ile112, Phe109, Val191, Tyr192, and His187 and a proximal polar region defined by residues Arg244, Glu108, and Asn171. Lastly, the larger GPR139 agonists (TC‐O 9311 and JNJ‐63533054), also share a less buried hydrophobic pocket defined by residues Val76, Phe79, Ile104, Val83, and Ile80. This common binding site was also previously identified by Norh et al^15^ Perhaps more importantly, we were able to identify mutations that rendered the receptor either more sensitive or less sensitive to the purported amino acid ligands. This should allow for the creation of transgenic animals that will require higher or lower levels of endogenous ligand to achieve activation, and enable more insight into the in vivo function of GPR139.

## 2 MATERIALS AND METHODS

### 2.1 Compounds

L‐Trp and L‐Phe were purchased from Sigma‐Aldrich (St. Louis, MO). JNJ‐63533054 was synthesized at Janssen Research & Development, LLC (San Diego, CA) as described in Dvorak et al^5^ (S)‐3‐bromo‐5‐chloro‐N‐(2‐oxo‐2‐((1‐phenylethyl)amino)ethyl)benzamide was used to prepare [^3^H]JNJ‐63533054 (24.7 Ci/mmol) via reduction of the bromide with tritium through a contract with Moravek Biochemicals (Brea, CA). The radiochemical purity of [^3^H]‐JNJ‐63533054 was determined to be 99.1% by high‐performance liquid chromatography (HPLC) analysis with radioactive flow detection. TC‐O 9311 (3,5‐dimethoxybenzoic acid 2‐[(1‐naphthalenylamino)carbonyl]hydrazide) was purchased from Tocris Bioscience (Bristol, UK).

### 2.2 Molecular cloning of GPR139

GPR139 from human was cloned from respective brain cDNAs as previously described in detail.^1^ N‐terminal V5‐tagged human GPR139 was created by adding a V5‐tag coding sequence (5′‐ CTC GAG GCC ACC ATG GGT AAG CCT ATC CCT AAC CCT CTC CTC GGT CTC GAT TCT ACG CGT GAA TTC GCC ACC‐3′) to the immediate 5′ upstream of the human GPR139 coding sequence. The gene was cloned into pCIneo (Promega, Wisconsin, MI) between Xho1 and Not1 sites and the insert region was sequenced (Eton Biosciences, San Diego, CA) to confirm the identities. The V5‐tag at the N‐terminus facilitates the detection of total and cell‐surface detection and measurement of GPR139 expression.

### 2.3 Calcium mobilization assay

HEK293 cells were transiently transfected with V5‐GPR139 (human) wild‐type or mutant clones using Fugene HD reagent (Promega, Madison, WI). Briefly, cells were grown to confluence in F‐12K culture media (Corning, Corning, NY) containing 10% fatal bovine serum (FBS), 1 × sodium pyruvate, 20 mmol L^−1^ HEPES. One day after transfection, cells were detached with 0.25% trypsin/2.25 mmol L^−1^ EDTA and resuspended in plating media F‐12K (Corning) containing 10% charcoal‐treated FBS, 1 × penicillin/streptomycin, 1 × sodium pyruvate, 20 mmol L^−1^ HEPES, and seeded at a density of 40 000 cells/well in poly‐D‐lysine‐coated, black‐walled, clear‐bottom 96‐well tissue culture plates and incubated overnight at 37°C, 5% CO_2_. Two days after transfection, cell culture medium was aspirated and cells were loaded with 1 × BD calcium loading dye (Becton Dickinson, Franklin Lakes, NJ) solution at 100 μL/well and incubated at 37°C, 5% CO_2_ for 45 minutes. Compound dilutions were prepared in Hank's balanced salt solution (HBSS) from 10 mmol L^−1^ dimethyl sulfoxide (DMSO) stocks while L‐Phe and L‐Trp dilutions were prepared from 30 mmol L^−1^ HBSS stocks. All compound additions (20 μL) were done on the Fluorometric Imaging Plate Reader Tetra (FLIPR‐Tetra; Molecular Devices, Sunnyvale, CA) and changes in fluorescence that reflect calcium mobilization were monitored at 1‐second intervals for 90 seconds, followed by 3‐second intervals for 60 seconds (excitation wavelength = 470‐495 nm, emission wavelength = 515‐575 nm). Data were exported as the difference between maximum and minimum fluorescence observed for each well. Results were calculated using nonlinear regression to determine agonist EC_50_ values (Graphpad Prism 7 software, San Diego, CA). *E*_max_ values are the percentage of the response elicited by the mutation clones compared with the one of wild type. Fold changes are determined by the ratio of the EC_50_ value of the mutation clones to the one of wild type.

### 2.4 Site directed mutation clones of GPR139

Site directed mutagenesis was performed using an overlapping PCR method. Specific primers (forward and reverse primers for each mutant as shown in Table S1) designed specific to the mutations were synthesized by Eton Biosciences. The N‐terminal V5‐tagged GPR139 DNA plasmid was used as the template for the PCR reactions. The 5′ end primer (5′ ATT CTT CTC GAG GCC ACC ATG GGT AAG CCT ATC‐3′) and the reverse primer for the mutant were used to PCR the 5′ end of the mutant gene while the forward primer of the mutant and the 3′ end primer (5′‐ ATG TCT GCG GCC GCT CAC GGG GAT ACT TTT ATA GGT TTT CCA TTT TTG TCA TAC TG‐3′) were used to PCR amplify the 3′ end of the mutant gene. The 5′ end and the 3′ end PCR products were mixed to serve as the template to synthesize the complete mutant gene by PCR using the 5′ end and the 3′ end primers described above. The PCR products were cloned into pCIneo between Xho1 and Not1 sites and the insert regions were sequenced to confirm the identities for each mutant gene.

### 2.5 Random mutation clones of GPR139

#### 2.5.1 Generation of GPR139 random mutation library

Random mutation clones were generated by PCR using N‐terminal V5‐tagged human GPR139 as the template. Forward primer (5′ ATT CTT CTC GAG GCC ACC ATG GGT AAG CCT ATC‐3′) and reverse primer (5′‐ ATG TCT GCG GCC GCT CAC GGG GAT ACT TTT ATA GGT TTT CCA TTT TTG TCA TAC TG‐3′) were used to amplify the gene for 40 cycles using Expand High Fidelity PCR System (Roche, IN) in the presence of 10% DMSO. The resulting PCR products were then cloned into the mammalian expression vector pCIneo (Promega). Twenty clones were sampled for the presence of mutations by DNA sequencing and 19 clones carried mutation(s). Plasmid DNAs from about 960 individual clones were isolated using 96‐well DNA preparation kits (Zyppy‐96 Plasmid miniprep kit, Zymo Research corp., Irvine, CA), according to the manufacturer's protocol.

#### 2.5.2 Characterization of GPR139 mutants from random mutagenesis

Expression of mutant GPR139 receptors was done by transfection of HEK293 cells grown in black 96‐well poly‐D‐lysine coated FLIPR plates using FUGENE‐HD (Promega) as the transfection reagent. Briefly, HEK293 cells were grown without antibiotics in black 96‐well poly‐D‐lysine coated FLIPR plates in F‐12K medium plus 10% FBS and 1 × sodium pyruvate at a density of 20 000 cells/well. One hundred nanograms per microliter of DNA and 3 μL of FUGENE‐HD were used for each well transfection. Triplicated cell plates were transfected for each 96‐well DNA plate. The wild‐type GPR139 DNA was used as the positive control and pCIneo plasmid without an insert was used as the negative control. Two days after transfection, the cell culture medium was replaced with HBSS buffer containing the Ca^2+^ dye and then incubated for 45 minutes at 37°C. A two‐addition FLIPR assay was used to characterize the mutant receptors with a 1^st^ addition of low concentration of L‐Trp (15 μmol L^−1^) and L‐Phe (30 μmol L^−1^) followed 3 minutes later by the addition of a high concentration of L‐Trp (1 mmol L^−1^) and L‐Phe (2 mmol L^−1^). The Ca^2+^ signal from the 1st addition, the 2nd addition, as well as the ratio of the signals from two additions were used to assess the relative response of the mutant receptors.

### 2.6 Total and surface V5‐GPR139 expression

V5‐tagged GPR139 protein expression was tested by ELISA assay using HEK293 cells expressing human V5‐GPR139 wild‐type and the mutant receptors. Mock‐transfected HEK293 cells served as the negative control. Transfected cells were plated in 96‐well poly‐D‐lysine plates at a cell density of 20 000 cells/well. For total GPR139 protein expression, the transfected cells were fixed using 10% formaldehyde in Dulbecco's phosphate buffered saline (DPBS, Hyclone, Pittsburgh, PA) and then treated with 1% Triton X‐100 (Sigma) in DPBS. Cells were blocked with 3% nonfat milk in 1% Triton X‐100 (Sigma) in DPBS and then incubated with 1 μg/ml of mouse monoclonal IgG2a Anti‐V5 antibody (Life Technologies, Carlsbad, CA) as the primary antibody. Second detection was carried out using goat anti‐mouse IgG, horseradish peroxidase (HRP) conjugate (30 ng/mL, Thermo Scientific, Waltham, MA) and developed with 3,3′,5,5′‐Tetramethylbenzidine (TMB) Substrate Reagent (BD Biosciences, San Jose, CA). Cell‐surface expression of V5‐GPR139 was measured by performing the ELISA assay described above without using Triton X‐100 as the penetrating reagent.

### 2.7 Radioligand binding assay

#### 2.7.1 Recombinant expression of wild‐type and mutant GPR139 receptors

Transient transfections of wild‐type and mutant GPR139 receptors were done in HEK293 cells using the Lipofectamine (Life Technologies) method according to manufacturer's instructions. Two days post‐transfection, cells were harvested in phosphate buffered saline (PBS) containing 10 mmol L^−1^ EDTA and spun down at 5000*g* at 4°C. Cell pellets were frozen at −80°C.

#### 2.7.2 Saturation binding assay

Membrane preparations from HEK293 cells transiently expressing the mutant and wild‐type human GPR139 receptors were incubated with eight concentrations of [^3^H]‐JNJ63533054 (specific activity 24.7 Ci/mmol) ranging from 6.25 to 400 nmol L^−1^ in assay buffer (50 mmol L^−1^ Tris‐HCl, 5 mmol L^−1^ EDTA pH 7.4) for 60 minutes at room temperature. Reactions were terminated by filtration through PEI‐coated GF/C filter plates (Perkin‐Elmer, Waltham, MA) followed by three washes with cold TE buffer. Filter plates were dried in a 60°C oven followed by addition of 45 μL scintillation fluid. Bound radioactivity was read on a Topcount scintillation counter. Nonspecific binding was determined with 10 μmol L^−1^ TC‐O 9311. Saturation binding data were analyzed using Graphpad Prism 7 software. The *B*_max_ and *K*_d_ values of the radioligand were determined using the one‐site binding (hyperbola) model.

#### 2.7.3 Competition binding assay

Membrane preparations from HEK293 cells transiently expressing the mutant and wild‐type human GPR139 receptor were incubated with seven concentrations of test compounds and [^3^H]‐JNJ63533054 at the predetermined *K*_d_ concentration for each wild‐type and mutant receptor for 1 hour at room temperature. Reactions were terminated by rapid filtration through PEI‐coated GF/C filter plates (Perkin‐Elmer), washed with ice‐cold TE buffer and dried in a 60°C oven for 30 minutes. Microscint‐0 was added to each well and bound radioactivity was read on a Topcount scintillation counter (Perkin‐Elmer, Waltham, MA). Nonspecific binding was determined with 10 μmol L^−1^ TC‐O 9311. Half‐maximal inhibitory concentrations (IC_50_s) of test compounds were calculated based on a nonlinear regression analysis in Graphpad Prism 6.0 (San Diego, CA). *K*_i_'s were determined according to the Cheng‐Prusoff equation.^1^

### 2.8 Molecular modeling

A homology model of hGPR139 was built using the nociceptin receptor (pdb 5DHG) as a template using Schrodinger Suites Release 2017‐03 (Schrödinger, LLC, New York, NY, 2017) Prime^16^, ^17^ module energy‐based method. The nociceptin receptor was chosen due to its high sequence similarity and *E*‐score when aligned (using BLAST) with GPR139. The initial homology model was used to run an Induced Fit Docking protocol^18^ with L‐Phe, L‐Trp, TC‐O 9311, and JNJ‐63533054 molecules. Docking site was chosen as the center of the orthosteric binding pocket. The typical GPCR orthosteric site was chosen due to mutation data suggesting residues in the orthosteric site can impact receptor function. An ensemble of docked structures was obtained from the induced fit docking protocol and was hand filtered and modified to generate a consensus GPR139 structure with ligands. This hand‐refined structure of GPR139 with JNJ‐63533054 was subjected to molecular dynamics simulation to further refine the structure.

Molecular dynamics (MD) of the GPR139 was performed using Desmond^19^ with the OPLS3 forcefield. Systems were setup using the system builder GUI in Maestro with POPC membrane model set to the transmembrane helices, SPC solvent model, a 12A water buffer, added ions to neutralize the system, and salt added to 0.15M NaCl concentration. The prepared systems were then subjected to 5 ns of restrained MD in which the helical backbone atoms were restraints of 10 kcal/mol. Restrained MD was performed at a temperature of 300 K, pressure of 1.01325 bar, with NPT ensemble. The default relaxation procedure (as implanted in Maestro) was used prior to simulation. Following the restrained MD simulation, all backbone restraints were removed and the system was further subjected to 25 ns of unrestrained simulation. The resulting unconstrained MD snapshots were clustered, and the clusters were used for Glide^19^ ensemble docking with all four ligands. The final GPR139 structure chosen had high docking scores for all four ligands (PDB file available in supplemental materials).

## 3 RESULTS

### 3.1 Response normalization strategy

**Figure 1:**
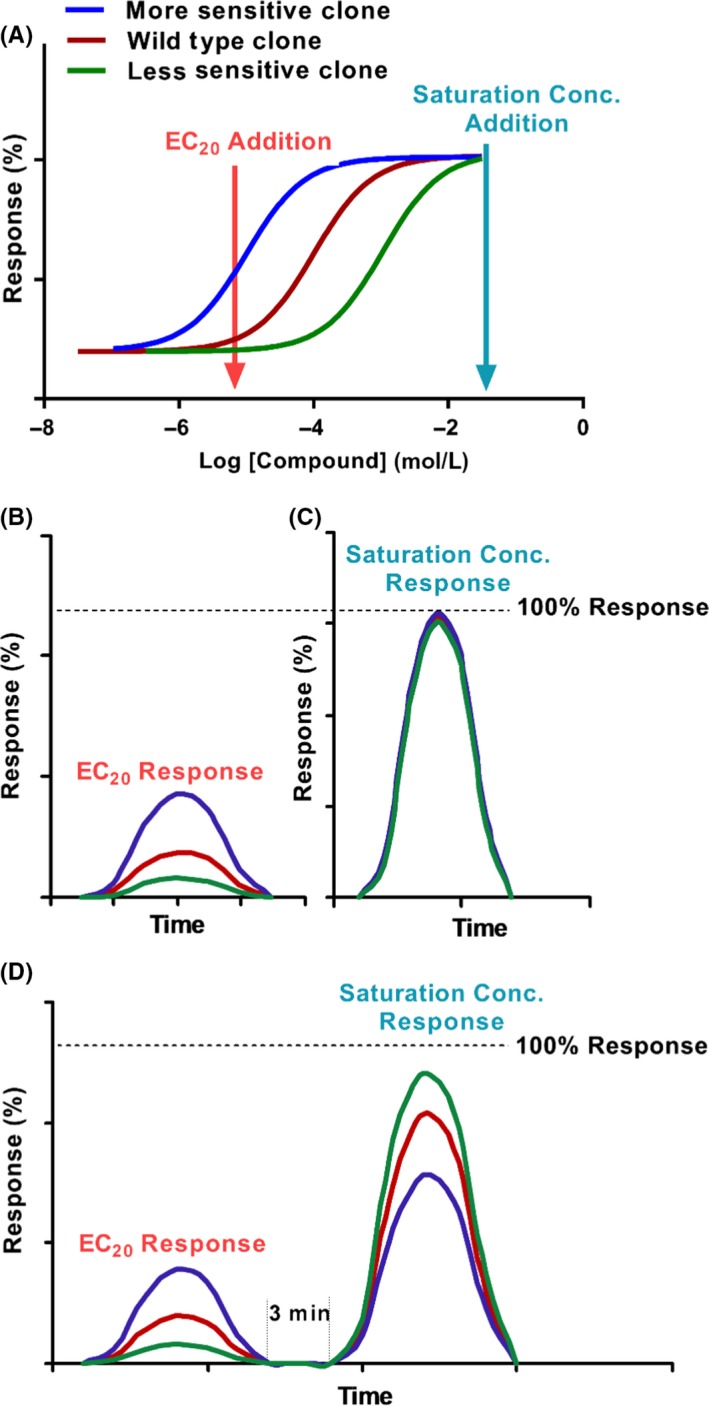
Illustration model of double additions and normalization strategy in calcium mobilization assay. (A) The EC_20_ dose of agonist and saturation dose of agonist were defined by GPR139 wild‐type dose response curve (B) Response curve of candidate mutation clones under the stimulation of wild‐type EC_20_ dose of agonist. (C) Response curve of candidate mutation clone under the stimulation of a wild‐type saturation dose of agonist. (D) Response curve of candidate mutation clone under the stimulation of double additions of first EC_20_ dose and then saturation dose of agonist. It is important to note that, due to different degree of receptor desensitization caused by the first addition of agonists, the response stimulated by the second addition with saturating concentration of agonists, will be lower than the response stimulated by the same saturating agonist without the first addition

In order to categorize the changes in sensitivity caused by random mutations of GPR139, all candidate clones were first prescreened by a calcium mobilization assay using a double addition normalization strategy (Figure 1). In essence, we devised this procedure to set each receptor as its own control for normalization in order to attempt to compare across various mutants. Figure 1 represents the theoretical percent (%) response of the random mutation clones in the FLIPR assay. The EC_20_ dose of agonist (mixture of 15 μmol L^−1^ of L‐Trp and 30 μmol L^−1^ of L‐Phe) and saturation dose of agonist (mixture of 1 mmol L^−1^ of L‐Trp and 2 mmol L^−1^ of L‐Phe) were defined by a GPR139 wild‐type dose response curve (Figure 1A).

When the candidate clone is stimulated by the EC_20_ dose of the agonist, a more sensitive clone will elicit a stronger response when compared to that of the wild type, while a less sensitive clone will elicit a weaker response (Figure 1B). These changes in response are due to the mutation induced shift in agonist potency as seen in Figure 1A. When the candidate clone is stimulated by a saturating dose of the agonist, all three types of clones will elicit *E*_max_ responses (Figure 1C).

When the “two additions” assay is applied, the candidate clone is first stimulated by the EC_20_ dose and 3 minutes later by the saturation dose of agonist. The response of EC_20_ dose is similar to that of single addition of EC_20_ dose illustrated in Figure 1B. This stimulation will lead to receptor desensitization and when the saturation dose addition is applied, a more sensitive clone will elicit a weaker response when compared to the wild type, instead a less sensitive clone will elicit a stronger response (Figure 1D). Consequently, the normalized percent response ratio of 1st/2nd addition is used to determine the sensitivity change of the candidate clones. The clone with a higher ratio (eg, 40%/60% = 2/3) compared to the wild type (eg, 20%/80% = 1/4) is considered as more sensitive clone and a lower ratio (eg, 10%/90% = 1/9) is considered as less sensitive clone. With this strategy, since each mutant receptor is self‐normalized, the response differences between different mutant clones caused by experimental conditions (eg, difference in cell numbers between wells, transfection efficiencies, protein expression levels, equipment reading differences, etc) will be normalized, and batches of data can be compared in this large‐scale screening. All the clones that show changes in sensitivity when compared to wild type are to be further characterized by dose response studies.

### 3.2 Characterization of human GPR139 receptor gain‐of‐function random mutations

We identified several human GPR139 receptor gain‐of‐function random mutation clones by calcium mobilization assay, in terms of their increased sensitivity to the putative endogenous ligands L‐Phe and L‐Trp (Table 1). The potency of the selective agonists TC‐O 9311 and JNJ‐63533054 with these mutation clones was also assessed in the assay (Table 1). We subcategorized Table 1 into single‐point mutation and multipoint mutation sections. Within each section, the mutations are listed in order from the most to the least potent mutation, in terms of the EC_50_ fold change to L‐Phe and then L‐Trp, with an arbitrary cut off value of 0.5, which means the EC_50_ value to L‐Phe or L‐Trp decreased at least by half, or in other words, the potency increased by at least 2X compared to the wild type. The potency to the selective agonists, TC‐O 9311 and JNJ‐63533054 were not significantly improved by most of the mutations that affected L‐Phe and L‐Trp (Table 1). Most of the gain‐of‐function mutations exhibited 22.6%‐73.4% reduction and 29.1% to 77.3% reduction in *E*_max_ regards to L‐Phe and L‐Trp response, respectively (Table 1). And a few of the gain‐of‐function mutations, including Val76Ala, IIe116Thr, Cys23Arg, and Asn93Asp, resulted in an increase of *E*_max_ to both endogenous ligands. In general, most of these gain‐of‐function mutations showed similar levels of total and cell‐surface protein expression levels (Table 1). Only a few double mutations exhibited moderate reduction (>50%) in both protein expressions (Table 1).

**Table 1:** Human GPR139 receptor gain‐of‐function random mutations summary

Section	Mutation	Location	L‐Phe		L‐Trp		TC‐O 9311		JNJ‐63533054		EC_50_ fold change				Total protein	Surface protein
			EC_50_ (μmol L^−1^)	*E*_max_ (%WT)	EC_50_ (μmol L^−1^)	*E*_max_ (%WT)	EC_50_ (n mol L^−1^)	*E*_max_ (%WT)	EC_50_ (nmol L^−1^)	*E*_max_ (%WT)	L‐Phe	L‐Trp	TC‐O 9311	JNJ‐3054	%WT	%WT
Single Point Mutations	V191A	TM5	15	65	14	62	31	61	67	59	0.15	0.16	0.81	2.86	73	57
	S267P	TM7	16	34	28	32	70	27	16	29	0.15	0.32	1.87	0.67	65	56
	V76A	TM2	18	176	38	232	18	192	11	199	0.17	0.42	0.48	0.46	172	165
	I116T	TM3	22	204	80	236	14	218	8	180	0.21	0.90	0.36	0.34	163	136
	F287Y	TM7	32	54	31	48	28	48	22	48	0.32	0.35	0.74	0.94	55	53
	C23R	N‐ter	32	107	84	122	33	112	12	129	0.32	0.95	0.88	0.51	127	119
	N93D	1st ECL	39	241	61	264	22	233	8	235	0.38	0.68	0.59	0.33	154	156
Multi Points Mutations	L249F, S267P	TM6, TM7	18	78	22	63	97	58	25	72	0.17	0.24	2.57	1.06	60	60
	I116T, N259S, D343G	TM3, 3rd ECL, C‐ter	25	33	32	28	32	30	42	29	0.25	0.36	0.84	1.78	40	39
	C23R, F78L	N‐ter, TM2	26	60	34	52	32	56	13	58	0.26	0.39	0.85	0.55	49	47
	D84N, N93D	TM2, 1st ECL	28	46	66	42	18	38	19	41	0.28	0.74	0.49	0.83	53	49
	P19L, V191A	N‐Ter, TM5	31	49	21	46	122	47	87	46	0.30	0.23	3.25	3.72	60	60
	D84G, K336Q	TM2, C‐ter	38	68	29	68	43	68	17	73	0.38	0.32	1.16	0.75	102	95
	V76A, I201V	TM2, TM5	39	72	38	71	56	67	36	71	0.39	0.43	1.48	1.52	72	69
	V191A, I229T	TM5, TM6	40	27	13	23	93	27	39	25	0.40	0.15	2.48	1.68	45	37
	T190A, P329Q	TM5, C‐ter	45	56	32	48	103	56	33	59	0.45	0.36	2.75	1.42	103	97
	WT		101	100	89	100	38	100	23	100	1.00	1.00	1.00	1.00	100	100

Agonist potency values were determined using a calcium mobilization assay in HEK 293 cells transiently transfected with mutated or wild‐type human GPR139. Within each section, the table is listed in an order from the most to the least potent mutations, in terms of the EC_50_ fold change to L‐Phe and then L‐Trp, with an arbitrary cut off value of 0.5. EC_50_ values are means from one experiment with duplicate or triplicate measurements. *E*_max_ values are expressed as the percentage of the response elicited by the wild‐type human GPR139. EC_50_ fold changes are expressed as a ratio, that EC_50_ of agonist of the mutation to the wild type. Total and surface protein expression values are expressed as the percentage of the mean absorbance value read against 450 nm of the wild‐type human GPR139, from one experiment with duplicate or triplicate measurements.

C‐Ter, C‐terminus; ECL, extracellular loop; ICL, intercellular loop; N‐Ter, N‐terminus; TM, transmembrane domain.

**Figure 2:**
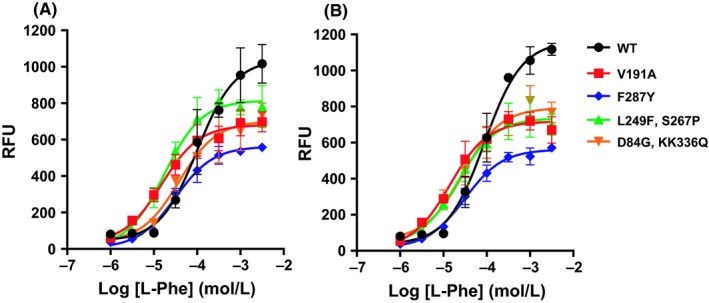
Dose response curves of the representative human GPR139 gain‐of‐function mutants to endogenous ligands (A) L‐Phe and (B) L‐Trp in calcium mobilization assay. Data shown as means ± SD with triplicate measurements for each point (n = 3)

In particular, we show that the single mutation Val191Ala, Ser267Pro, Val76Ala, and IIe116Thr resulted in 5.7 to 6.6‐fold increase and 2.4 to 6.3‐fold increase in L‐Phe and L‐Trp potency, respectively (Table 1). The effect of these mutations on ligand binding affinity were characterized using a saturation binding assay with [^3^H]‐JNJ‐63533054 (Table 2). We show that Val191Ala and Ile116Thr exhibited 2.6 and 4.3‐fold decrease in the ligand *K*_d_, respectively, with the other two mutations not significantly affected. Val191Ala and Val76Ala showed about 2‐fold increase in *B*_max_ while Ser267Pro showed a 3.7‐fold decrease in *B*_max_, with Ile116Thr not significantly affecting it. The decrease of *B*_max_ with Ser267Pro mutation may partially account for the decrease of its *E*_max_ in the FLIPR assay (33% of wild type). The binding properties of L‐Phe, L‐Trp, TCO‐9311, and JNJ‐63533054 were characterized using a competitive inhibition assay using the [^3^H]‐JNJ‐63533054 as the radioligand. In general, the *K*_i_ value of the ligands were not significantly affected by these mutations (Table 2). The dose response curves to L‐Phe and L‐Trp of the representative gain‐of‐function mutants are shown in Figure 2A and B.

**Table 2:** Representative human GPR139 receptor gain‐of‐function random mutations radioligand binding assays summary

Mutation	*K*_d_ (nmol L^−1^)	*B*_max_ (fg/mg protein)	*K*_d_ fold change	*B*_max_ fold change	*K*_i_ (μmol L^−1^)		*K*_i_ (nmol L^−1^)		*K*_i_ fold change			
					L‐Phe	L‐Trp	TC‐O 9311	JNJ‐3054	L‐Phe	L‐Trp	TCO‐9311	JNJ‐3054
V191A	3 ± 1	8061 ± 2703	0.23	2.24	643 ± 320	1072 ± 465	56 ± 11	19 ± 8	0.73	1.08	0.53	0.56
S267P	11 ± 5	1011 ± 531	0.83	0.27	554 ± 140	682 ± 171	322 ± 164	46 ± 15	0.72	0.74	2.85	1.43
V76A	22 ± 11	6713 ± 1721	1.63	1.93	360 ± 121	626 ± 78	44 ± 16	43 ± 17	0.41	0.70	0.39	1.29
I116T	5 ± 3	3170 ± 1442	0.38	0.86	875 ± 146	1015 ± 215	56 ± 18	26 ± 10	0.98	1.11	0.50	0.81
WT	13 ± 3	3674 ± 1554	1.00	1.00	990 ± 426	1019 ± 364	110 ± 23	37 ± 24	1.00	1.00	1.00	1.00

Concentration binding of [^3^H]‐JNJ‐63533054 were determined using a saturation binding assay in HEK293 cells transiently transfected with mutated or wild‐type human GPR139. *K*_d_ (nmol L^−1^) and *B*_max_ (fg/mg protein) values are means ± SD (n = 3). *K*_d_ and *B*_max_ fold changes are expressed as a ratio, that EC_50_ of agonist of the mutation to the wild type. Competition of [^3^H]‐JNJ63533054 binding by L‐Phe, L‐Trp, TC‐O 9311, and JNJ‐63533054 were determined using a competitive inhibition assay in COS‐7 cells transiently transfected with mutated or wild‐type human GPR139 wild‐type human GPR139. Inhibition equilibrium constants (*K*_i_) values are means ± SD (n = 3). *K*_i_ fold changes are expressed as a ratio, that EC_50_ of agonist of the mutation to the wild type.

### 3.3 Characterization of human GPR139 receptor reduction‐of‐function random mutations

We also identified a number of human GPR139 receptor mutations that resulted in reduction‐of‐function as measured by ligand‐induced calcium mobilization (Table 3). Again, we subcategorized Table 3 into single point mutation and multipoint mutation sections. Within each section, the mutations are listed in an order from the most to the least potent mutations, in terms of the EC_50_ fold change to L‐Phe and then L‐Trp, with an arbitrary cut off value of 2.0, which means the EC_50_ value to L‐Phe or L‐Trp increased by at least 2X, or in other words, the potency decreased at least by half compared to the wild type. Most of these mutations also exhibited a significant decrease in potency (at least by half compared to wild type) to the selective agonists, TC‐O 9311 and JNJ‐63533054 (Table 3). Also, most of these reduction‐of‐function mutations exhibited moderate to significant reduction in *E*_max_ regards to all four ligands responses. (Table 3). In terms of total and cell‐surface protein expression levels, most of these mutants showed more than 50% decrease in both protein expression levels (Table 3).

**Table 3:** Human GPR139 receptor reduction‐of‐function random mutations summary

Section	Mutation	Location	L‐Phe		L‐Trp		TC‐O 9311		JNJ‐63533054		EC_50_ Fold Change				Total Protein	Surface Protein
			EC_50_ (μmol L^−1^)	*E*_max_ (%WT)	EC_50_ (μmol L^−1^)	*E*_max_ (%WT)	EC_50_ (n mol L^−1^)	*E*_max_ (%WT)	EC_50_ (n mol L^−1^)	E_max_ (%WT)	L‐Phe	L‐Trp	TC‐O 9311	JNJ‐3054	%WT	%WT
Single Point Mutations	C286R	TM7	ND	N/A	ND	N/A	450	21	50	24	N/A	N/A	14.38	7.21	51	46
	K225E	TM6	ND	N/A	ND	N/A	179	111	31	118	N/A	N/A	5.71	4.39	22	40
	Y33C	TM1	1695	83	623	57	160	79	55	76	21.61	10.71	5.11	7.87	44	65
	T278I	TM7	1205	45	1356	35	225	27	27	46	15.36	23.30	7.18	3.80	44	51
	L86P	TM2	725	11	451	15	116	22	57	90	9.24	7.75	3.69	8.19	22	28
	L41S	TM1	539	121	241	104	145	133	45	132	6.88	4.15	4.62	6.40	14	23
	N203D	TM5	497	136	416	112	74	154	19	151	6.34	7.15	2.36	2.72	10	17
	L249H	TM6	491	58	336	32	89	54	24	68	6.26	5.77	2.83	3.49	26	38
	F231S	TM6	386	29	416	28	111	29	31	32	4.92	7.15	3.53	4.42	21	32
	S62P	TM2	297	92	195	82	62	95	23	237	3.78	3.36	1.99	3.25	20	31
	L249P	TM6	202	43	190	42	87	42	23	132	2.57	3.27	2.78	3.26	53	37
Multi Points Mutations	I128N F237S	TM3, TM6	ND	N/A	2188	59	339	66	56	61	N/A	37.59	10.83	8.03	21	31
	I205T K225E	TM5, ICL3	ND	N/A	1617	77	110	89	24	98	N/A	27.78	3.51	3.46	32	50
	K147E F283S	ICL2, TM7	1223	58	530	42	465	64	57	71	15.59	9.11	14.84	8.08	14	25
	M96V Y136C	ECL1, ICL2	572	47	283	50	80	45	20	50	7.29	4.87	2.54	2.86	33	48
	R57G I288V	ICL1, C‐Ter	523	49	330	48	340	67	51	279	6.66	5.66	10.86	7.30	42	44
	V148A I229T	TM4, TM6	520	54	300	50	155	53	24	54	6.63	5.15	4.96	3.43	21	37
	I45V S222F	TM1, ICL3	435	136	155	141	55	143	10	154	5.55	2.66	1.74	1.45	25	39
	I288N A302T	C‐Ter, C‐Ter	333	60	232	48	147	64	32	73	4.25	3.99	4.71	4.53	25	34
	M96V F282Y	ECL1, TM7	228	44	115	38	87	53	23	52	2.90	1.97	2.78	3.26	30	42
	I45T T226A	TM1, ICL3	164	53	102	48	82	53	16	51	2.09	1.76	2.62	2.28	28	37
	WT		78	100	58	100	31	100	7	100	1.00	1.00	1.00	1.00	100	100

Agonist potency values were determined using a calcium mobilization assay in HEK 293 cells transiently transfected with mutated or wild‐type human GPR139. Within each section, the table is listed in an order from the most to the least potent mutations, to the EC_50_ fold change to L‐Phe and then L‐Trp, with an arbitrary cut off value of 2.0. EC_50_ values are means from one experiment with duplicate or triplicate measurements. *E*_max_ values are expressed as the percentage of the response elicited by the wild‐type human GPR139. EC_50_ fold changes are expressed as a ratio, that EC_50_ of agonist of the mutation to the wild type. Total and surface protein expression values are expressed as the percentage of the mean absorbance value read against 450 nm of the wild‐type human GPR139, from one experiment with duplicate or triplicate measurements.

C‐Ter, C‐terminus; ECL, extracellular loop; ICL, intercellular loop; N/A, not applicable; ND, not determined as the concentration response curve did not reach plateau; N‐Ter, N‐terminus; TM, transmembrane domain.

**Figure 3:**
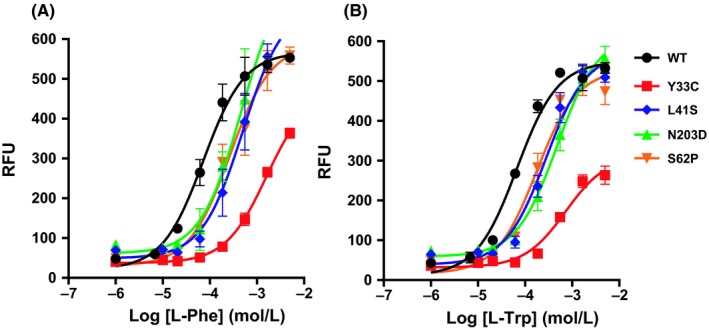
Dose response curves of the representative human GPR139 reduction‐of‐function mutants to endogenous ligands (A) L‐Phe and (B) L‐Trp in calcium mobilization assay. Data shown as means ± SD with triplicate measurements for each point (n = 3)

In particular, the single mutation Cys286Arg, Lys225Glu and the double mutations Ile128Asn, Phe237Ser resulted in a significant decrease in L‐Phe and L‐Trp potency, which was beyond the detection limit of the FLIPR assay (Table 3, Figure 3). All three mutations also exhibited 5.7 to 14.4‐fold and 4.4 to 8.0‐fold decrease in TCO‐9311 and JNJ‐63533054 potency, respectively (Table 3). These results confirmed the expression of these mutated receptors on the cell membrane, which was further proven by the cell‐surface protein expression assay, though decreased compared to the wild type (Table 3). The binding affinity of selected four single mutants was also characterized by the radioligand binding assays. Leu86Pro resulted in a 16.8‐fold increase in *K*_d_ and a 2.2‐fold decrease in *B*_max_ (Table 4). This mutation also resulted in a significant reduction in the ability of L‐Phe, L‐Trp, TCO 9311, and JNJ‐63533054 to compete for binding, which was revealed by a 42.6 to 363.2‐fold decrease in *K*_i_ value of the ligands in the competitive inhibition assay (Table 4). In terms of the other three selected mutations, the *K*_d_ values were not significantly affected. All three mutations showed 1.7 to 6.2‐fold decrease in *B*_max_ (Table 4), which is in consistent with their decreased cell‐surface expression (Table 3). The binding properties of L‐Phe, L‐Trp, TC‐O 9311. and JNJ‐63533054 (*K*_i_) were not significantly affected by these mutations (Table 4). The dose response curves to L‐Phe and L‐Trp of the representative reduction‐of‐function mutants are shown in Figure 3A and B.

**Table 4:** Representative human GPR139 receptor reduction‐of‐function random mutations radioligand binding assays summary

Mutation	*K*_d_ (nmol L^−1^)	*B*_max_ (fg/mg protein)	*K*_d_ fold change	*B*_max_ fold change	*K*_i_ (μmol L^−1^)		*K*_i_ (nmol L^−1^)		*K*_i_ fold change			
					L‐Phe	L‐Trp	TC‐O 9311	JNJ‐3054	L‐Phe	L‐Trp	TC‐O 9311	JNJ‐3054
C286R	12 ± 9	1187 ± 376	1.54	0.51	543 ± 21	517 ± 57	45 ± 11	25 ± 12	1.09	1.03	0.83	0.56
T278I	4 ± 4	1400 ± 439	0.57	0.59	306 ± 62	186 ± 28	42 ± 5	13 ± 4	0.59	0.37	0.44	0.54
L86P	142 ± 65	1066 ± 593	16.80	0.45	3231 ± 526	3833 ± 721	5952 ± 789	9755 ± 5589	139.27	42.59	363.16	78.13
L41S	5 ± 1	381 ± 146	0.59	0.16	591 ± 223	501 ± 70	39 ± 3	16 ± 2	1.12	0.99	0.58	0.50
WT	9 ± 2	2359 ± 297	1.00	1.00	523 ± 150	519 ± 158	81 ± 21	30 ± 9	1.00	1.00	1.00	1.00

Concentration binding of [^3^H]‐JNJ‐63533054 was determined using a saturation binding assay in HEK293 cells transiently transfected with mutated or wild‐type human GPR139. *K*_d_ (nmol L^−1^) and *B*_max_ (fg/mg protein) values are means ± SD (n = 3). *K*_d_ and *B*_max_ fold changes are expressed as a ratio, that EC_50_ of agonist of the mutation to the wild type. Competition of [^3^H]‐JNJ63533054 binding by L‐Phe, L‐Trp, TC‐O 9311 and JNJ‐63533054 were determined using a competitive inhibition assay in COS‐7 cells transiently transfected with mutated or wild‐type human GPR139 wild‐type human GPR139. Inhibition equilibrium constants (*K*_i_) values are means ± SD (n = 2). *K*_i_ fold changes are expressed as a ratio, that EC_50_ of agonist of the mutation to the wild type.

### 3.4 Characterization of human GPR139 receptor loss‐of‐function random mutations

Apart from the gain‐ and reduction‐of‐function mutations, we also identified a list of human GPR139 receptor loss‐of‐function random mutations. These mutations resulted in the lack of responses to all four tested ligands at the highest concentrations applied. We further categorized these loss‐of‐function mutations into two sub groups. Some of these mutants exhibited proper, though decreased, total and cell‐surface expression levels (Table 5), indicating the loss of function by the mutations was caused by loss of potency to the ligands. The other mutants showed limited total and cell‐surface protein expressions (Table S2), attributing the loss of their function to the limited receptor expression. For the sake of the data completeness, we also listed the random mutations which resulted in no significant potency change to L‐Phe and L‐Trp (EC_50_ change less than 100% compared to the wild type, data not shown), all presented with proper levels of total and cell‐surface expression compared to the wild type (Table S3).

**Table 5:** Human GPR139 receptor loss‐of‐function random mutations with protein expression summary

Mutation	Location	Total protein	Surface protein
		%WT	%WT
H113Y P142Q	TM3, ICL2	93	109
P238T V311C	TM6, C‐Ter	50	42
S197P F200Y	TM5, TM5	46	43
L77F N331S	TM2, C‐ter	44	32
I248V Y285C	TM6, TM7	35	49
N281D Y285H	TM7, TM7	29	31
WT		100	100

Total and surface protein expression values are expressed as the percentage of the mean absorbance value read against 450 nm of the wild‐type human GPR139, from one experiment with duplicate or triplicate measurements.

C‐Ter, C‐terminus; ICL, intercellular loop; TM, transmembrane domain.

### 3.5 Characterization of human GPR139 receptor site directed mutations

As transmembrane domain 3 (TM3) is considered as one of the common core domains involved in ligand binding and activation in most receptors that respond to small organic molecules,^20^ we also carried out a screening of human GPR139 receptor TM3 site directed mutations, from Ile103 to Val130. All residues were mutated to Ala individually and the potency to all four ligands was assessed in the calcium mobilization assay (Table S4). We report that TM3 site‐directed mutations did not result in any gain‐of‐function revealed by none of the EC_50_ to the ligands decreased at least by half, compared to the wild type. On the other hand, some of the mutations resulted in reduction‐of‐function, as revealed by their loss of response (beyond detection limit) to endogenous ligands L‐Phe and L‐Trp or even to the selective agonists TC‐O 9311 and JNJ‐63533054, including Phe109Ala, Ile112Ala, His113Ala, Trp117Ala, Ile118Ala, Asp125Ala, and Arg126Ala. All the site directed mutations exhibited proper levels of both total and cell‐surface expressions. Of note is Phe109Ala, which is one of the mutations that caused a complete loss of function to all four compounds. This result is consistent with an earlier report indicating Phe109 is highly important for these ligands ability to bind and mutation to alanine resulted in a complete loss of function.^15^ Nøhr et al^15^ also indicated Asn271 as another common binding site shared by these ligands and a mutation from a hydrophilic residue Asn to a hydrophobic residue Ala also resulted in a complete loss of function. In our case, we identified Asn271Ser change through random mutation studies, which mutated between two hydrophilic residues, and did not cause significant functional changes (Table S3). Ile112 forms the bottom of the orthosteric site and mutation to an Ala would impact ligand binding. And lastly Asp125 and Arg126 are a part of the DRY motif at the end of TM3 that is involved in G‐protein binding, mutations to these residues disrupt the ability of the GPCR to bind with the G‐protein.

### 3.6 Homology model of human GPR139 receptor

**Figure 4:**
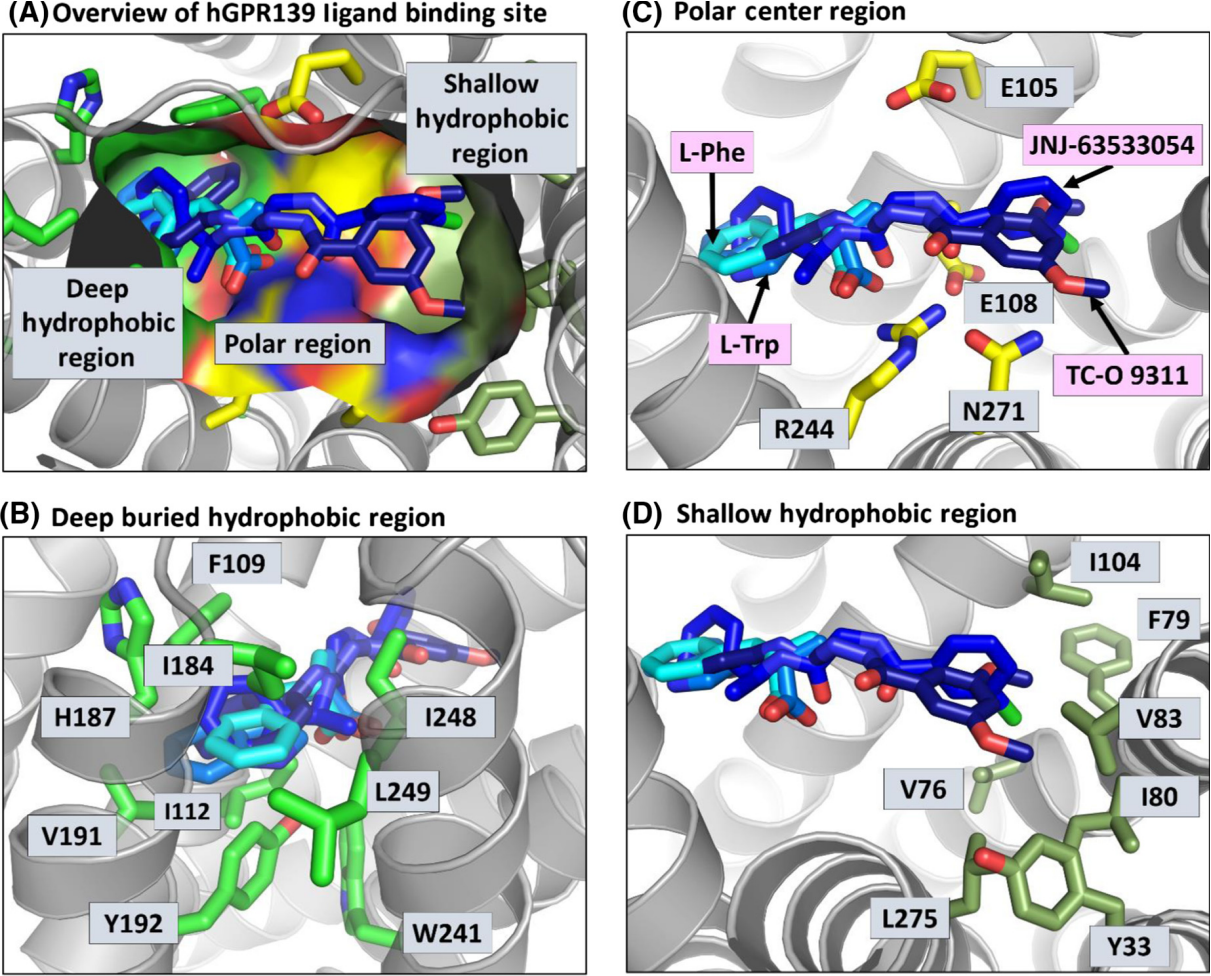
Homology model of hGPR139 with four docked ligands. (A) An overview of the ligand binding site with surface representation is shown color coded based on region with the deep hydrophobic region in green, polar region in yellow, and shallow hydrophobic region in olive. Detail views of the residues involved in (B) deep hydrophobic region, (C) polar region, and (D) shallow hydrophobic region are shown with the residues in sticks representation in green, yellow, and olive respectively. Each ligand is indicated in panel (C) and the color goes from darkest blue for the largest compound to the lightest blue for the smallest compound (TC‐O 9311, JNJ‐63533054, L‐Trp, L‐Phe)

**Figure 5:**
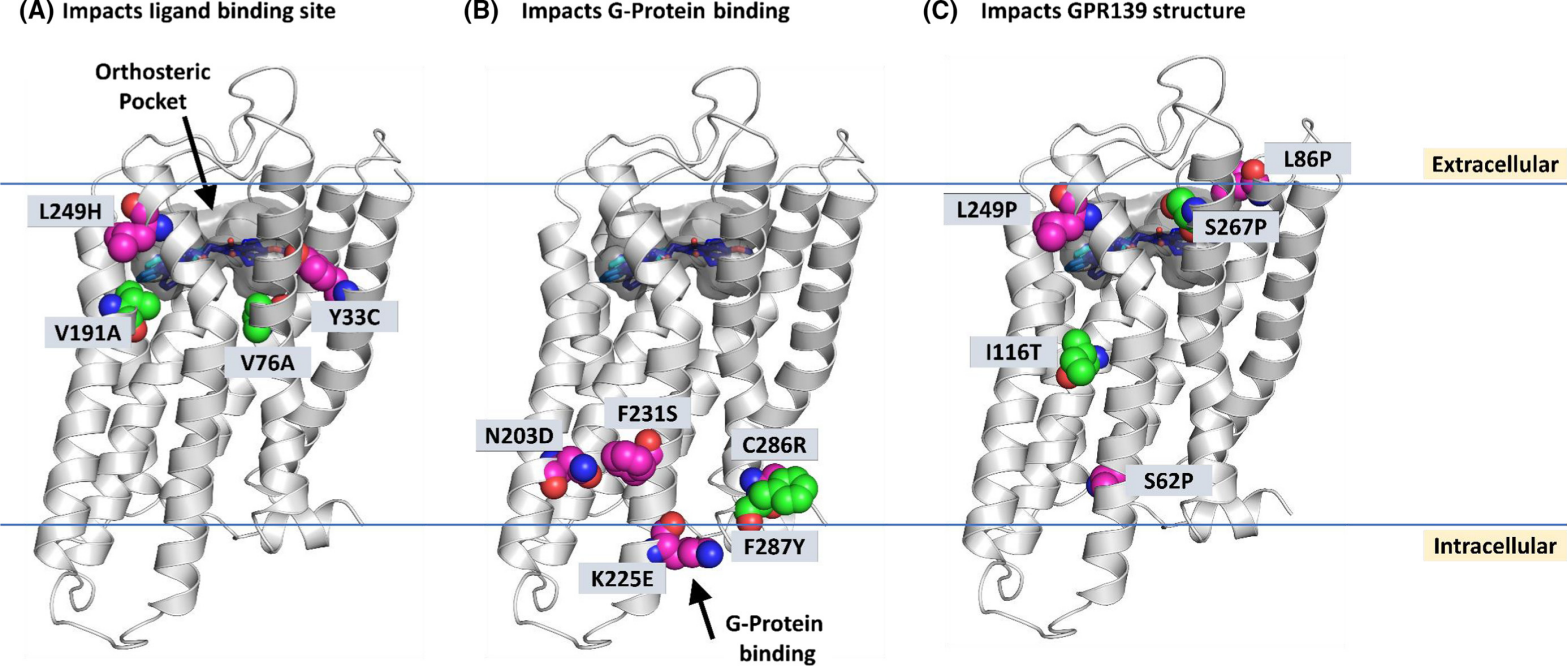
Selected single‐point gain‐of‐function and reduction‐of‐function mutations mapped onto a homology model of hGPR139. Homology model of hGPR139 (gray cartoon) with TC‐O 9311, JNJ‐63533054, L‐Trp, L‐Phe (various blue shaded sticks with the same coloring order in Figure 4) docked into the orthosteric pocket (transparent gray surface). Gain‐of‐function mutations are shown with carbon atoms as green spheres, and reduction‐of‐function mutations in with carbon atoms as pink spheres, nitrogen atoms are shown as blue and oxygen atoms as red spheres, respectively. Identified mutations are grouped by their impact on (A) orthosteric ligand binding site, (B) G‐protein binding area, and (C) GPR139 secondary or tertiary structure

Finally, all the structure‐activity data described above are incorporated into a homology model of hGPR139 with all four ligands presented. An overview of hGPR139 ligand binding site with surface representation is shown in Figure 4. A common binding site for TC‐O 9311 and JNJ‐63533054 and endogenous amino acids L‐Trp and L‐Phe (Figure 4A) was previously identified by Norh et al,^15^ with a deeply buried hydrophobic region between Phe109, His187, and Trp241 (Figure 4B) and a polar region defined by Asn271, Arg244, and Glu108 (Figure 4C). The ligands were described as positioning themselves in a way that their aromatic moieties fit in the deep hydrophobic region while their polar or charged regions bind to Asn271 and Arg244. The homology modeling done in our study suggested a very similar binding site with the additional identification of a less buried, shallow hydrophobic pocket that is shared by TC‐O 9311 and JNJ‐63533054 comprised of residues Tyr33, Val76, Phe79, Ile80, Val83, Ile104, and Leu275 (Figure 4D). We also mapped single point gain‐of‐function and reduction‐of‐function mutations onto the homology model (Figure 5). The possible mechanisms of these mutations affecting structure‐activity relationship are addressed in the discussion. As a summary, locations of mutations identified as gain‐of‐function (Tables 1 and 2), reduction‐of‐function (Table 3 and 4) and loss‐of‐function with protein expression (Table 5) are shown in Figure S5A, S5B and S5C, respectively.

## 4 DISCUSSION

In the present study, we identified a series of human GPR139 mutations which result in a gain, reduction, or loss of function. To further characterize these single and multiresidue mutations, we mapped their positions onto a homology model of GPR139 in order to interpret their impact on both ligand binding and receptor activation.

Several single‐point mutations were identified to residues forming the orthosteric pocket (Figure 5A). These include two gain‐of‐function mutations Val191Ala and Val76Ala (Tables 1 and 2) and two reduction‐of‐function mutations Leu249His and Tyr33Cys (Table 3). These residues along with the previously identified residues from Norh et al help refine our understanding of the orthosteric ligand binding pocket of hGPR139. The binding pocket consists of a deep buried hydrophobic region and a shallow hydrophobic region linked together by a polar region (Figure 4A). This matches closely with the pharmacophoric features of the two larger compounds TC‐O 9311 and JNJ‐63533054, which consists of two hydrophobic aromatic moieties linked together by a polar linker, while the smaller endogenous amino acids contain a subset of this pharmacophore, a single hydrophobic aromatic moiety linked to a polar group.

Another set of mutations (Figure 5B) were identified which likely impact G‐protein binding as they are all located on the intracellular half of the GPCR near where G‐protein binds.^21^ These include three reduction‐of‐function mutations Asn203Asp, Phe231Ser, and Lys225Glu (Table 3) which reside on helix 5 and 6, two helices that are known to structurally shift upon binding of G‐proteins.^22^ Additionally, Phe287Tyr, and Cys286Arg, a gain‐of‐function (Table 1) and a reduction‐of‐function (Table 3) mutation located at the junction of helix 7 with the terminal helix 8 also residing in an area in close proximity to G‐protein binding.

A third set of mutations (Figure 5C) were identified which impact the transmembrane helices structure which in turn could change the shape of the orthosteric pocket or the ability for the G‐ protein to bind. These include reduction‐of‐function mutations Leu249Pro, Ser62Pro, and Leu86Pro (Table 3) and gain‐of‐function mutations Ile116Thr and Ser267Pro (Table 1). All but one of these mutations are to a proline residue. Proline residues are known to break helical secondary structure thus altering the helical nature of the transmembrane helices. Ile116Thr is the only exception, it is found to be both the gain‐of‐function mutation in the calcium mobilization (Table 1) and the radioligand binding (Table 2) assays. It sits near the orthosteric pocket but is not involved in forming the ligand binding site. However, it sits just under Val191Ala (identified as residue that impacts ligand binding site, Figure 5A). A mutation of a hydrophobic isoleucine to a polar threonine, likely impacts the relative placement of Val191 and thus impacts the structure of the orthosteric site.

There are two additional sets of mutations identified which could have a structural impact on the function of GPR139. The first set of mutations, Thr278Ile and Leu41Ser Figure S5(B), were identified as reduction‐of‐function in both calcium mobilization and radioligand binding assays (Tables 3 and 4). These residues reside in a region that is known to form a water channel important for GPCR activation.^23^ In particular, the Thr278Ile mutation would impact the water channel itself by changing from a polar threonine residue to a hydrophobic isoleucine residue. Leu41Ser sits just proximal to Thr278Ile but would not be involved in formation of the channel. However, a mutation from a large hydrophobic leucine to a small polar serine likely impacts the placement of the Thr278 residue changing the shape of the water channel. The second set of mutations, Asn93Asp and Cys23Arg are two extracellular loop mutations (Figure S5A) that were identified as gain‐of‐function in the calcium mobilization assay (Table 1). At first glance these residues would appear to be in a location that would not impact the structure of the GPCR, the ability of the ligands to bind orthostatic pocket, nor the ability of G‐proteins to bind. However, movement of the transmembrane helices is known to be required for GPCR activation and given the connectedness of the extracellular region to the transmembrane helices, mutations to this region could influence the orientation of the transmembrane helices and thus modulate receptor activation.^24^

Further, we identified several multipoint loss‐of‐function mutations with proper protein expression (Table 5, Figure S5C). Although it is harder to pinpoint the exact structural changes resulting from multipoint mutations, three of the loss‐of‐function mutations are either proline mutations to nonproline residues or vice versa, including Pro142Gln at the end of TM2, Pro238Thr in the middle of TM6, and Ser197Pro in the middle of TM5. As discussed previously, proline residues are known to break helical structures and any mutations involving prolines could impact secondary structure. Additionally, two loss‐of‐function multipoint mutations involving Tyr285 were identified. Tyr285 is located at the bottom of TM7 in a region where G‐protein binding occurs and where other mutations (Phe287Tyr and Cys286Arg) were found to also impact GPR139 function.

In conclusion, our study demonstrated several critical structural motifs in human GPR139 receptor activity. These lines of information not only facilitate the rational design of novel potent and selective agonists and antagonists of GPR139, but also guide the design of transgenic animal models (carrying the gain‐of‐function or reduction‐of‐function point mutations) for better understanding the physiological function of GPR139 in the future. In particular, traditional knockout models may lead to a compensatory phenotype by the system. While in comparison, the gain‐in function transgenic animal may provide a different angle exploring the biological function of receptors, which are activated by their natural ligands with relatively high EC_50_ values under normal conditions.

## AUTHOR CONTRIBUTIONS

*Participated in research design:* Wang, Shih, Wu, Bonaventure, Lovenberg and Liu;

*Conducted experiments:* Lee, Shih, Kuei, Nepomuceno, Wennerholm, Fan and Liu;

*Performed data analysis:* Wang, Lee, Shih, Kuei, Nepomuceno, Wennerholm, Fan and Liu;

*Wrote or contributed to the writing of the manuscript:* Wang, Lee, Shih, Nepomuceno, Wu, Bonaventure, Lovenberg and Liu.

## DISCLOSURE

None declared.
