# Supplementary material for: Mutagenesis of GPR139 reveals ways to create gain or loss of function receptors
Source: Pharmacol Res Perspect. 2019 Feb 7;7(1):e00466. doi: 10.1002/prp2.466 (PMC6367278; doi:10.1002/prp2.466)
Supplement: Supplementary file 1 [file PRP2-7-e00466-s001.docx]

**Title: Mutagenesis of GPR139 reveals ways to create gain or loss of function receptors**

**Supplemental Data**

**Table S1**

**Forward and reverse primers designed for site directed mutation clones of human GPR139 receptor**

| **Mutation** | **Forward primer (5’ to 3’)** | **Reverse primer (5’ to 3’)** |
| --- | --- | --- |
| **I103A** | CAGGTCCCCGACAAGgcaATAGAAGTGCTGGAA | TTCCAGCACTTCTATtgcCTTGTCGGGGACCTG |
| **I104A** | GTCCCCGACAAGATCgcaGAAGTGCTGGAATTC | GAATTCCAGCACTTCtgcGATCTTGTCGGGGAC |
| **E105A** | GTCCCCGACAAGATCATAgcaGTGCTGGAATTC | GAATTCCAGCACtgcTATGATCTTGTCGGGGAC |
| **V106A** | GACAAGATCATAGAAgcaCTGGAATTCTCATCC | GGATGAGAATTCCAGtgcTTCTATGATCTTGTC |
| **L107A** | AAGATCATAGAAGTGgcaGAATTCTCATCCATC | GATGGATGAGAATTCtgcCACTTCTATGATCTT |
| **E108A** | ATCATAGAAGTGCTGgcaTTCTCATCCATCCAC | GTGGATGGATGAGAAtgcCAGCACTTCTATGAT |
| **F109A** | ATAGAAGTGCTGGAAgcaTCATCCATCCACACC | GGTGTGGATGGATGAtgcTTCCAGCACTTCTAT |
| **S110A** | GAAGTGCTGGAATTCgcaTCCATCCACACCTCC | GGAGGTGTGGATGGAtgcGAATTCCAGCACTTC |
| **S111A** | GTGCTGGAATTCTCAgcaATCCACACCTCCATA | TATGGAGGTGTGGATtgcTGAGAATTCCAGCAC |
| **I112A** | CTGGAATTCTCATCCgcaCACACCTCCATATGG | CCATATGGAGGTGTGtgcGGATGAGAATTCCAG |
| **H113A** | GAATTCTCATCCATCgcaACCTCCATATGGATT | AATCCATATGGAGGTtgcGATGGATGAGAATTC |
| **T114A** | TTCTCATCCATCCACgcaTCCATATGGATTACT | AGTAATCCATATGGAtgcGTGGATGGATGAGAA |
| **S115A** | TCATCCATCCACACCgcaATATGGATTACTGTA | TACAGTAATCCATATtgcGGTGTGGATGGATGA |
| **I116A** | TCCATCCACACCTCCgcaTGGATTACTGTACCG | CGGTACAGTAATCCAtgcGGAGGTGTGGATGGA |
| **W117A** | CACACCTCCATATGGgcaACTGTACCGTTAACC | GGTTAACGGTACAGTtgcCCATATGGAGGTGTG |
| **I118A** | CACACCTCCATATGGgcaACTGTACCGTTAACC | GGTTAACGGTACAGTtgcCCATATGGAGGTGTG |
| **T119A** | ACCTCCATATGGATTgcaGTACCGTTAACCATT | AATGGTTAACGGTACtgcAATCCATATGGAGGT |
| **V120A** | TCCATATGGATTACTgcaCCGTTAACCATTGAC | GTCAATGGTTAACGGtgcAGTAATCCATATGGA |
| **P121A** | ATATGGATTACTGTAgcaTTAACCATTGACAGG | CCTGTCAATGGTTAAtgcTACAGTAATCCATAT |
| **L122A** | TGGATTACTGTACCGgcaACCATTGACAGGTAT | ATACCTGTCAATGGTtgcCGGTACAGTAATCCA |
| **T123A** | ATTACTGTACCGTTAgcaATTGACAGGTATATC | GATATACCTGTCAATtgcTAACGGTACAGTAAT |
| **I124A** | ACTGTACCGTTAACCgcaGACAGGTATATCGCT | AGCGATATACCTGTCtgcGGTTAACGGTACAGT |
| **D125A** | GTACCGTTAACCATTgcaAGGTATATCGCTGTC | GACAGCGATATACCTtgcAATGGTTAACGGTAC |
| **R126A** | CCGTTAACCATTGACgcaTATATCGCTGTCTGC | GCAGACAGCGATATAtgcGTCAATGGTTAACGG |
| **Y127A** | TTAACCATTGACAGGgcaATCGCTGTCTGCCAC | GTGGCAGACAGCGATtgcCCTGTCAATGGTTAA |
| **I128A** | ACCATTGACAGGTATgcaGCTGTCTGCCACCCG | CGGGTGGCAGACAGCtgcATACCTGTCAATGGT |
| **V130A** | GACAGGTATATCGCTgcaTGCCACCCGCTCAAG | CTTGAGCGGGTGGCAtgcAGCGATATACCTGTC |
| **V191A** | ATCCACTGCTTCACCgctTACCTGGTGCCCTGC | GCAGGGCACCAGGTAagcGGTGAAGCAGTGGAT |

**Table S2**

**Human GPR139 receptor loss-of-function random mutations without protein expression summary**

Total and surface protein expression values are expressed as the percentage of the mean absorbance value read against 450 nm of the wildtype human GPR139, from one experiment with duplicate or triplicate measurements.

| Mutation | Location | Total Protein | Surface Protein |
| --- | --- | --- | --- |
|  |  | **%WT** | **%WT** |
| P195S | TM5 | 11 | 17 |
| F27S C131Y | N-Ter, ICL2 | 12 | 17 |
| I91T | ECL1 | 19 | 18 |
| L77P I80L | TM2, TM2 | 13 | 18 |
| Y32C P42L | TM1, TM1 | 21 | 23 |
| P29L T234A | N-Ter, TM6 | 10 | 20 |
| C38R | TM1 | 14 | 18 |
| R244C | TM6 | 19 | 19 |
| P142S | ICL2 | 10 | 16 |
| T234I H264Y | TM6, ECL3 | 19 | 27 |
| L276P | TM7 | 15 | 21 |
| I104V M272V | ECL1, TM7 | 17 | 24 |
| L37P S178P | TM1, ECL2 | 12 | 20 |
| L252P L273P | ECL3, TM7 | 12 | 18 |
| L36S I198T | TM1, TM5 | 17 | 20 |
| L35P R126W | TM1, TM3 | 16 | 17 |
| W185R I186V | TM5, TM5 | 15 | 22 |
| S110P V120A | TM3, TM3 | 12 | 19 |
| L51P E172G | TM1, ECL2 | 12 | 19 |
| S140P | ICL2 | 17 | 23 |
| H137Y N281S | ICL2, TM7 | 15 | 25 |
| L240P A242V | TM6, TM6 | 12 | 18 |
| L51P | TM1 | 12 | 17 |
| T190P | TM5 | 12 | 18 |
| L240P | TM6 | 12 | 18 |
| H5Y Y192H | N-Ter, TM5 | 20 | 21 |
| WT |  | 100 | 100 |

TM, transmembrane domain; ECL, extracellular loop; ICL, intercellular loop; N-Ter, N-terminus;

**Table S3**

**Human GPR139 receptor random mutations with no activity change (L-Trp or L-Phe EC50 change less than 100%) summary**

Total and surface protein expression values are expressed as the percentage of the mean absorbance value read against 450 nm of the wildtype human GPR139, from one experiment with duplicate or triplicate measurements.

| Mutation | Location | Total Protein | Surface Protein |
| --- | --- | --- | --- |
|  |  | **%WT** | **%WT** |
| S34G | TM1 | 123 | 107 |
| L14P, V30M | N-ter, TM1 | 103 | 102 |
| L202S, H264R | TM5, 2nd ECL | 102 | 101 |
| T155A, S267Y, V311I | TM3, TM7, C-ter | 95 | 93 |
| I118V | TM3 | 94 | 88 |
| A72T | TM2 | 87 | 83 |
| G224R | ICL3 | 68 | 82 |
| F79S | TM2 | 63 | 57 |
| N271S | TM7 | 54 | 54 |
| S12C V194A | N-Ter, TM5 | 48 | 74 |
| I74T | TM2 | 47 | 44 |
| L39F, V83E | TM1, TM2 | 42 | 40 |
| I206T A228G | TM5, TM6 | 42 | 62 |
| F237S A274V | TM6, TM7 | 37 | 38 |
| T340C | C-Ter | 32 | 36 |
| V83G | TM2 | 31 | 41 |
| T223A F231S | ICL3, TM6 | 29 | 32 |
| Y63H P325S | ICL1, C-Ter | 28 | 31 |
| T223M | ICL3 | 27 | 36 |
| L41I S115A | TM1, TM3 | 26 | 34 |
| V148A | TM4 | 25 | 33 |
| L183P | ECL2 | 23 | 28 |
| T114A S235P | TM3, TM6 | 15 | 22 |
| WT |  | 100 | 100 |

TM, transmembrane domain; ECL, extracellular loop; ICL, intercellular loop; N-Ter, N-terminus; C-Ter, C-terminus;

**Table S4**

**Human GPR139 receptor site directed mutations summary**

Agonist potency values were determined using a calcium mobilization assay in HEK 293 cells transiently transfected with mutated or wildtype human GPR139. EC_50_ values are means from one experiment with duplicate or triplicate measurements. E_max_ values are expressed as the percentage of the response elicited by the wildtype human GPR139. EC_50_ fold changes are expressed as a ratio, that EC_50_ of agonist of the mutation to the wildtype. Total and surface protein expression values are expressed as the percentage of the mean absorbance value read against 450 nm of the wildtype human GPR139, from one experiment with duplicate or triplicate measurements.

| Mutation | Location | L-Phe | | L-Trp | | TC-O 9311 | | JNJ-63533054 | | EC_50_ Fold Change | | | | Total Protein | Surface Protein |
| --- | --- | --- | --- | --- | --- | --- | --- | --- | --- | --- | --- | --- | --- | --- | --- |
|  |  | **EC_50_ (μM)** | **E_max_ (%WT)** | **EC_50_ (μM)** | **E_max_ (%WT)** | **EC_50_ (nM)** | **E_max_ (%WT)** | **EC_50_ (nM)** | **E_max_ (%WT)** | **L-Phe** | **L-Trp** | **TC-O 9311** | **JNJ-3054** | **%WT** | **%WT** |
| I103A | TM3 | 88 | 28 | 57 | 31 | 53 | 35 | 10 | 32 | 1.23 | 1.05 | 1.16 | 0.81 | 60 | 62 |
| I104A | TM3 | 125 | 70 | 105 | 68 | 51 | 71 | 6 | 74 | 1.74 | 1.92 | 1.11 | 0.47 | 58 | 60 |
| E105A | TM3 | 501 | 153 | 219 | 161 | 29 | 165 | 11 | 152 | 6.98 | 4.00 | 0.63 | 0.85 | 44 | 65 |
| V106A | TM3 | 104 | 73 | 78 | 71 | 41 | 72 | 8 | 71 | 1.45 | 1.43 | 0.89 | 0.66 | 51 | 56 |
| L107A | TM3 | 224 | 50 | 257 | 46 | 98 | 37 | 30 | 39 | 0.93 | 1.30 | 1.25 | 1.22 | 65 | 75 |
| E108A | TM3 | 277 | 84 | 152 | 73 | 37 | 75 | 11 | 67 | 1.15 | 0.77 | 0.48 | 0.44 | 82 | 88 |
| F109A | TM3 | ND | N/A | ND | N/A | ND | N/A | ND | N/A | N/A | N/A | N/A | N/A | 90 | 103 |
| S110A | TM3 | 402 | 79 | 251 | 65 | 92 | 65 | 23 | 62 | 1.68 | 1.27 | 1.18 | 0.95 | 80 | 97 |
| S111A | TM3 | 634 | 104 | 404 | 82 | 138 | 106 | 35 | 108 | 2.65 | 2.04 | 1.75 | 1.43 | 95 | 76 |
| I112A | TM3 | ND | N/A | ND | N/A | 533 | 23 | 217 | 26 | N/A | N/A | 6.78 | 8.84 | 59 | 62 |
| H113A | TM3 | ND | N/A | ND | N/A | 268 | 46 | 69 | 40 | N/A | N/A | 3.40 | 2.82 | 67 | 68 |
| T114A | TM3 | 721 | 74 | 791 | 68 | 108 | 71 | 23 | 69 | 3.01 | 3.99 | 1.37 | 0.93 | 105 | 109 |
| S115A | TM3 | 437 | 60 | 450 | 53 | 107 | 75 | 45 | 61 | 1.82 | 2.27 | 1.36 | 1.83 | 104 | 98 |
| I116A | TM3 | 78 | 221 | 42 | 222 | 49 | 242 | 21 | 199 | 1.09 | 0.77 | 1.06 | 1.71 | 102 | 84 |
| W117A | TM3 | ND | N/A | ND | N/A | 1371 | 7 | 54 | 7 | N/A | N/A | 29.88 | 4.32 | 46 | 36 |
| I118A | TM3 | ND | N/A | ND | N/A | 172 | 44 | 32 | 57 | N/A | N/A | 3.74 | 2.59 | 65 | 59 |
| T119A | TM3 | 121 | 100 | 88 | 98 | 43 | 99 | 8 | 107 | 1.69 | 1.60 | 0.93 | 0.65 | 76 | 72 |
| V120A | TM3 | 187 | 111 | 63 | 115 | 63 | 116 | 21 | 95 | 2.61 | 1.15 | 1.38 | 1.66 | 66 | 54 |

ND, not determined as the concentration response curve did not reach plateau; N/A, not applicable; TM, transmembrane domain

**Table S4 (Continued)**

| Mutation | Location | L-Phe | | L-Trp | | TC-O 9311 | | JNJ-63533054 | | EC_50_ Fold Change | | | | Total Protein | Surface Protein |
| --- | --- | --- | --- | --- | --- | --- | --- | --- | --- | --- | --- | --- | --- | --- | --- |
|  |  | **EC_50_ (μM)** | **E_max_ (%WT)** | **EC_50_ (μM)** | **E_max_ (%WT)** | **EC_50_ (nM)** | **E_max_ (%WT)** | **EC_50_ (nM)** | **E_max_ (%WT)** | **L-Phe** | **L-Trp** | **TC-O 9311** | **JNJ-3054** | **%WT** | **%WT** |
| P121A | TM3 | 156 | 73 | 51 | 61 | 108 | 60 | 27 | 59 | 2.17 | 0.94 | 2.36 | 2.18 | 102 | 113 |
| L122A | TM3 | 427 | 128 | 283 | 129 | 91 | 141 | 33 | 152 | 5.96 | 5.17 | 1.98 | 2.64 | 74 | 91 |
| T123A | TM3 | 172 | 292 | 70 | 261 | 31 | 264 | 13 | 251 | 2.40 | 1.28 | 0.68 | 1.02 | 92 | 115 |
| I124A | TM3 | 171 | 104 | 53 | 94 | 107 | 95 | 37 | 93 | 2.38 | 0.97 | 2.32 | 2.94 | 97 | 80 |
| D125A | TM3 | ND | N/A | ND | N/A | 142 | 32 | 44 | 31 | N/A | N/A | 3.10 | 3.52 | 59 | 40 |
| R126A | TM3 | ND | N/A | ND | N/A | ND | N/A | ND | N/A | N/A | N/A | N/A | N/A | 73 | 82 |
| Y127A | TM3 | 228 | 158 | 127 | 135 | 75 | 139 | 21 | 134 | 3.18 | 2.33 | 1.64 | 1.68 | 64 | 64 |
| I128A | TM3 | 167 | 159 | 101 | 134 | 64 | 149 | 28 | 140 | 2.33 | 1.85 | 1.39 | 2.21 | 68 | 76 |
| V130A | TM3 | 145 | 125 | 85 | 104 | 81 | 117 | 19 | 111 | 2.02 | 1.55 | 1.76 | 1.53 | 64 | 71 |
| WT |  | 72 | 100 | 55 | 100 | 46 | 100 | 12 | 100 | 1.00 | 1.00 | 1.00 | 1.00 | 100 | 100 |

**Figure. S5**

**
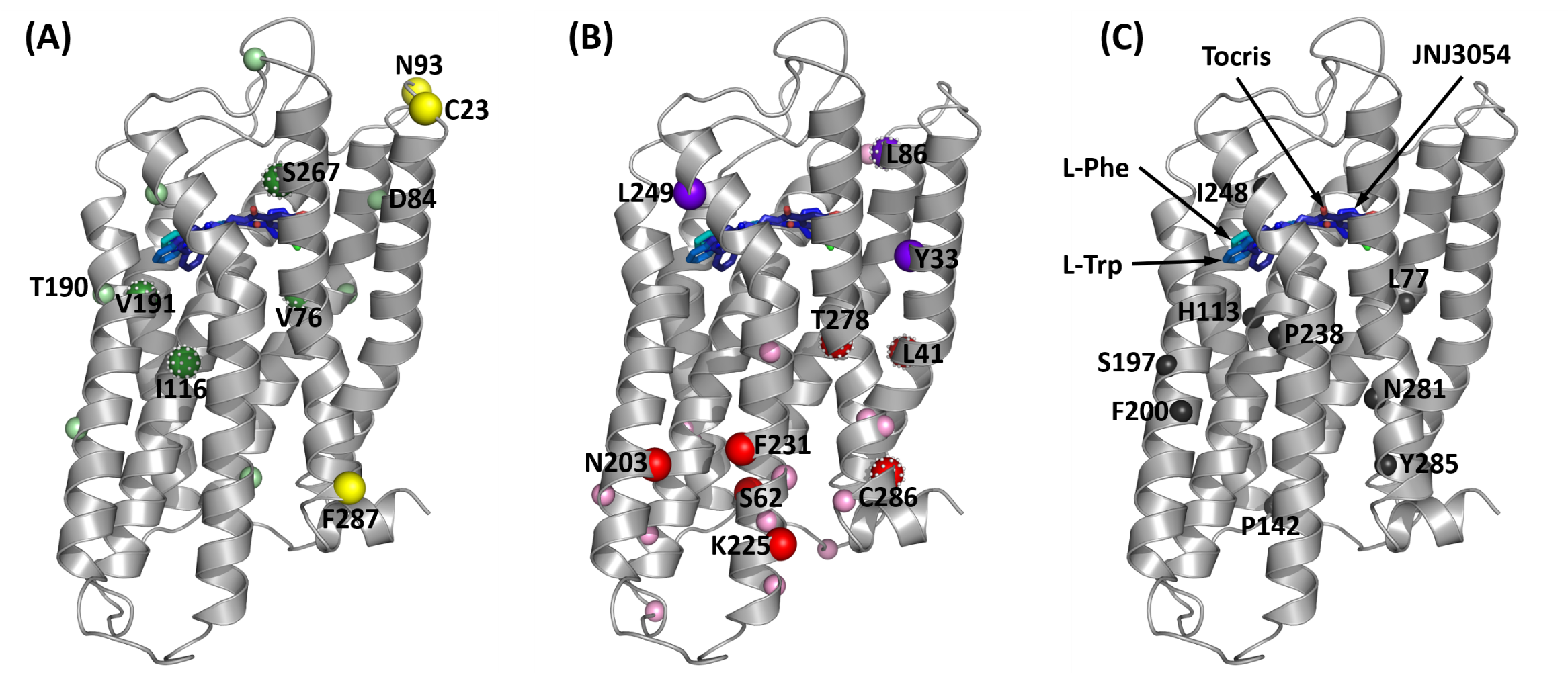
**

**Figure S5. Homology model of hGPR139 (shown in gray cartoon representation) with all four ligands.** Location of mutations identified as gain in function, reduction in function, and loss of function are shown in panels (**A**), (**B**), and (**C**), respectively. (**A**) Gain-of-function mutations identified via single point mutations in both the radioligand binding assay and calcium mobilization assay (green spheres with white dots) are all adjacent to the orthosteric pocket. Additional single point gain-of-function mutations identified by calcium mobilization assay (yellow) are far from the orthosteric pocket. Multi-points gain-of-function mutations are shown as small pale spheres. (**B**) Reduction-of-function mutations identified by calcium mobilization assay are shown as red and purple spheres, while mutations also identified by in the radioligand binding assay have additional small pale spheres. Those residues colored in purple are proximal to the orthosteric pocket. Multi-points reduction-of-function mutations are shown as small pink spheres. (**C**) Complete loss-of- function double-points mutations are shown as black spheres. Each ligand is indicated in panel (**C**) and the color goes from darkest blue for the largest compound to the lightest blue for the smallest compound (TC-O 9311, JNJ-63533054, L-Trp, L-Phe).
